# Activation of the flexor reflex for gait training in chronic stroke patients

**DOI:** 10.3389/fneur.2026.1794512

**Published:** 2026-07-27

**Authors:** Aida Sehle, Joachim Liepert

**Affiliations:** 1 Lurija Institute, Allensbach, Germany; 2 Kliniken Schmieder, Allensbach, Germany

**Keywords:** chronic stroke, flexor reflex, gait training, overground training, treadmill training

## Abstract

Mobility limitations are a common and quality-of-life–reducing consequence of stroke. A variety of therapeutic approaches exist to restore or improve walking ability, including electrical stimulation methods. One technique that has rarely been applied in this context is the electrical elicitation of the flexor reflex. This pilot study investigated whether gait training combined with supportive flexor reflex activation in patients in the chronic phase after stroke improves walking speed and walking distance. Five individuals with chronic stroke (mean 3.8 years post-stroke) completed two baseline assessments (T0 and T1), followed by a 5-week training phase comprising 15 therapy sessions (treadmill training and overground walking at moderate intensity). Post-intervention assessments were performed immediately after training (T2) and at follow-up visits 3 months (T3) and 12 months (T4) later. Outcomes included the 10-meter walk test and the 2-min walk test, each performed with and without flexor reflex activation. Additional assessments included comfortable and maximal treadmill walking speed, the User Satisfaction Evaluation Questionnaire, and video recordings of gait patterns. Significant differences between walking with and without flexor reflex activation were observed in both the 10-meter walk test and the 2-min walk test at pre- and post-training assessments. Performance in both tests improved significantly after the training phase, and these improvements remained detectable at T3. The User Satisfaction Evaluation Questionnaire indicated a high level of satisfaction with the device. Blinded video analysis by a physiotherapist indicated improved gait quality (greater safety and speed) both after training and during flexor reflex activation. This study shows for the first time in ambulatory chronic stroke outpatients, who were not enrolled in inpatient neurological rehabilitation, that gait training combined with flexor reflex activation improves walking speed and walking distance. The intervention resulted in sustained improvements in gait parameters, which were also reflected in qualitative gait changes. However, the respective contributions of gait training and flexor reflex activation cannot be distinguished and should be examined in a future randomized controlled trial.

## 1 Introduction

A significant impairment following a stroke is the limitation of walking ability and thus mobility, initially affecting about 80% of patients (1). Approximately 30% of stroke patients do not achieve independent walking (2). Those who do achieve independent walking often suffer from reduced gait speed and impaired endurance (3). Exercise-induced improvements in walking abilities are associated with an improved quality of life (4).

To improve walking ability, various therapeutic options are available depending on the degree of impairment. These options include an appropriate gait training program as well as the use of various technologies. Methods include treadmill gait training (5, 6), overground gait training (7), circuit training (8), robotic therapy (9–11) and action observation training (12). Additionally, a popular method is electrical stimulation treatment (13), as utilized in functional electrical stimulation.

In this study, a different approach was chosen. This technique represents an infrequently utilized method in gait rehabilitation of stroke patients. In contrast, this method has been used since the 1980s in patients with spinal cord injury, both in diagnostic and rehabilitative contexts (14–17). It involves the electrical activation of the flexor reflex (FR) at a precise moment within the walking cycle. Triggering of the FR is accomplished through electrical stimulation at the foot’s sole, activating a spinal reflex pathway that leads to simultaneous hip, knee, and ankle flexion. This technique of stimulating the FR by electrically targeting the sole of the foot is suggested as a potential treatment for enhancing gait recovery post-stroke (18–23). In particular, for patients with stroke-related weakness of the hip flexors, the FR can contribute to better step initiation, thereby facilitating the forward movement of the paretic leg.

A recently published systematic review and meta-analysis summarized the available evidence on withdrawal reflex stimulation during gait training after stroke (23). Krewer et al. (18) included three studies and reported that electrical stimulation of the nociceptive withdrawal reflex during walking was associated with significantly improved walking speed compared with gait training without reflex activation. However, the authors concluded that the certainty of evidence was low, mainly because only a small number of studies was available and the investigated patient populations were predominantly severely affected. Therefore, the generalizability of the findings remains limited, and further controlled studies are needed.

In the previously published studies, electrical stimulation of the FR while walking has predominantly been applied to patients in the subacute phase after a stroke (18–20). Therapeutic use in chronic stroke patients has only been documented in the form of a single case report (21). To date, no study has investigated the effects of multi-session gait training combined with activation of the FR during walking in chronic stroke patients. In Sehle et al. (22), 25 patients with subacute or chronic stroke were examined in a single-session randomized design. Walking performance was assessed with and without Incedo® using the 10-meter walk test, the 2-min walk test, and gait analysis. Significant but modest improvements were observed with Incedo® in walking speed and walking distance, whereas gait parameters remained largely unchanged except for step length. However, this study investigated the immediate orthotic effect of FR activation only and did not examine the effects of repeated gait training sessions. Notably, patients in these earlier studies were also generally severely affected, with scores on the Functional Ambulation Category (FAC) ranging from 0 to 2. In addition, these patients were undergoing neurological rehabilitation, and the training was integrated into their regular therapy schedule. In contrast, participants in the current study were not enrolled in inpatient neurological rehabilitation but attended the clinic specifically for training sessions alongside their everyday routines. This setting allows for a clearer assessment of the intervention’s effectiveness. Moreover, these patients were ambulatory, with a Functional Ambulation Category (FAC) score of 3–4, indicating independent walking ability.

This study was designed to evaluate the short term and long term effects of intensive gait training combined with reflex-based electrical stimulation in chronic stroke patients.

## 2 Methods

### 2.1 Subjects

In order to recruit patients, 155 physiotherapy practices within a 30 km radius of Kliniken Schmieder Allensbach, Germany, were informed about the planned intervention study via letter or email. Information about the study was also published on the clinic’s own Facebook and Instagram accounts. A review of 230 files of former stroke patients from the clinic was conducted. The criteria for selecting participants were as follows: patients over 18 years of age, those diagnosed with persistent hemiparesis due to a stroke occurring more than 6 months before, those who could communicate, understand tasks, and follow directions, those who could independently walk for a minimum of 10 meters with or without assistive devices. Patients were required to be able to tolerate electrostimulation and show a visible response to stimulation while walking. Exclusion criteria included a prior history of neurological illnesses or psychiatric conditions, lack of compliance, epilepsy, patients with heart pacemakers, severe heart or lung diseases, cancer, pregnancy, and skin lesions in the area where the electrode was to be positioned.

The present study was approved by the ethics committee of the University of Constance, Germany, and it was registered in the German Clinical Trials Register (DRKS00036294). The objectives and procedures to be performed in the study were fully understood by the participants, and all participants provided informed consent before inclusion in the study. All participants provided written informed consent. This study was conducted according to the ethical principles of the Declaration of Helsinki.

Altogether, 41 patients were identified and contacted by phone or email. Eight patients agreed to participate in a preliminary investigation. During this appointment, the Incedo® (Nordic NeuroSTIM ApS, Aalborg, Denmark) electrical stimulation device was introduced to the patients, applied, and tested to assess how well the affected leg responded to stimulation. If the response to stimulation of the affected leg while sitting was sufficient, patients were also asked to try walking with the device activated. In one patient, a very weak response was observed while sitting, and no response was detected during stimulation of the FR while walking. Another patient was highly sensitive to the electrical stimulation and found it very unpleasant, even at lower intensity levels. A third patient had a very unstable ankle joint, which posed a risk of ankle sprain (supination trauma) even with stimulation of the FR during walking.

The remaining five patients showed a good response to stimulation of the FR, both while sitting and walking, and were confident in their ability to complete the training program. Finaly, these five patients participated in the study.

The group consisted of four men and one woman, with a mean age of 65.7 ± 11 years (mean ± standard deviation) and a mean post-stroke duration of 3.8 ± 1.2 years. Three patients had right-sided and two left-sided hemiparesis. In everyday life, three participants used a foot drop orthosis, whereas two did not; two walked with a cane and one with a rollator.

### 2.2 Technical setup and functionality of the Incedo® system

The Incedo® system was used for eliciting the FR. The system consists of an impulse generator, electrodes, and a sensor. The electrodes are attached to the patient’s sole of the foot. The sensor is placed in the patient’s shoe. As soon as the sensor recognizes the initiation of a step, a signal is sent to the impulse generator, which produces an electrical stimulation through the electrode. The FR is activated to produce movements of the hip, knee, and foot joints during the swing phase, thereby supporting the swing phase of gait.

The Incedo® device operates with the following specifications:

- Pulse: Balanced biphasic.
- Pulse Pattern: Four 15 Hz pulse trains consisting of 5 pulses each, delivered at 200 Hz.
- Pulse Duration: 1 ms.
- Intensity: Adjustable from 0 to 50 mA.
- Maximal Voltage: 270 V.
- Maximal Load: 5400 *Ω*.

The Incedo® device is current-controlled to ensure precise and consistent stimulation. Its foot sensor detects pressure changes to trigger stimulation without delay once the foot is lifted. The FR itself includes an inherent delay of approximately 140 ms, timed to align optimally with the gait cycle.

Calibration was performed with the patient seated. Stimulation intensity was increased in 0.5 mA steps until a strong motor response was observed or discomfort occurred. Patients were instructed to report any discomfort. Walking with the device followed, during which the stimulation was adjusted: increased until uncomfortable, then reduced by 0.5 mA to a tolerable level.

### 2.3 Assessments

In total, patients were assessed five times over the course of the study: at baseline (T0), 5 weeks later as a second baseline (T1), after 5 weeks of therapy (T2), following a 12-week follow-up period (T3), and 1 year after the intervention (T4). One patient was lost to follow-up at the one-year mark. The two baseline assessments (T0 and T1) were conducted to evaluate the stability of the outcome measures.

The following assessments were performed at all measurement time points: a 10-meter walk test (10mWT) (24) with and without the Incedo® device (measured in seconds), a 2-min walk test (2minWT) (25) with and without the device (measured in meters). The 10mWT represents speed-related aspects of walking, while the 2minWT reflects endurance-related aspects. These parameters are the primary outcome measures. The assessments were performed in a standardized order. First, participants completed the 10mWT without the Incedo® device, followed by the 10mWT with the Incedo® device. After a 5-min rest period, which was included to minimize potential carry-over effects of electrical stimulation on subsequent testing, participants completed the 2minWT first without and then with the Incedo® device.

Furthermore, comfortable and maximum walking speeds on a treadmill, the User Satisfaction Evaluation Questionnaire, and video recordings of patients’ gait patterns were defined as secondary outcome measures.

Before and after the 5-week training period, both the comfortable and the maximum walking speed on a treadmill, measured in kilometers per hour (km/h), were assessed. These assessments were performed on the treadmill on the first and the last training day, respectively. First, the comfortable walking speed was tested, followed by the maximum walking speed. To determine the maximum walking speed on the treadmill, the treadmill speed was continuously increased from the comfortable walking speed until the maximum walking speed was reached. Patients were expected to maintain their maximum walking speed for 30 s without stumbling or losing balance (26). This duration was chosen to keep the burst short enough to reduce the influence of fatigue, while still providing sufficient time for individuals with stroke to reach their peak treadmill walking speed (27, 28).

After the training period (T2), and at the end of the assessments, the patients were asked to complete the User Satisfaction Evaluation Questionnaire (USEQ) (29). This standardized questionnaire assesses users’ subjective satisfaction with a medical device or system. It consists of seven items rated on a 5-point Likert scale (1 = “not at all” to 5 = “very much”). The questions cover aspects such as enjoyment of use, perceived effectiveness, control over the system, clarity of information, discomfort experienced, perceived usefulness for lower-limb rehabilitation, and willingness to continue using the system. The purpose of administering the USEQ was to evaluate the acceptance and subjective user experience of the Incedo® system as part of the training program.

The video recordings were obtained after the gait assessments, following a 15-min rest period. Video recordings of the patients’ gait patterns were made with and without the Incedo® device to capture qualitative changes in gait. These recordings were performed before (T1) and after the 5-week training period (T2) and evaluated by a physiotherapist who was blinded to the conditions. Therefore, the physiotherapist was unaware of which videos were recorded before or after the training, and similarly, was unaware of when the Incedo® device was active or inactive during walking. The physiotherapist’s task was to determine which videos were taken before and after training and to identify when the patients were walking with the activated Incedo® device versus without activation. The physiotherapist provided a commentary for each video, justifying her assessment and giving reasons for their evaluation.

Additionally, sensory testing of plantar foot sensation and muscle strength assessment of the affected lower limb were performed prior to the initiation of the training intervention. These tests were conducted before the assessments. Somatosensory function was assessed by comparing touch sensation at the sole of the affected foot with that of the less affected foot. The sensation of the less affected foot was defined as 100%. Patients were then asked to estimate the sensation of the affected foot as a percentage of the less affected side. The results were expressed as the percentage ratio between the affected and less affected foot, as described by Sehle et al. (22). Muscle strength assessment of the affected lower limb was performed using the Medical Research Council (MRC) grading scale, which ranges from 0 to 5 (30). The primary aim of this assessment was to identify sensory impairments as well as the weakest muscle groups on the affected side in order to characterize the extent and distribution of motor impairment.

### 2.4 Combined gait training with Incedo®

Training at a low to moderate intensity level is typically advised during the chronic stage of stroke recovery (31). This corresponds to 40–70% of VO₂ reserve, 55–80% of maximum heart rate, or a perceived exertion rating between 11 and 14 on a 20-point scale (32).

In this study, the gait training program consisted of 15 sessions over a period of 5 weeks, combining treadmill training (8 sessions) and overground training (7 sessions), all conducted with the Incedo® device. This combined method was selected to provide patients with a versatile, varied, and challenging training program aimed at improving gait safety, walking speed, and endurance.

The combination of treadmill and overground gait training constitutes a suitable intervention for stroke patients, as it allows for the targeted improvement of distinct gait parameters and supports the transfer of gains achieved on the treadmill to functional overground walking (28). Given that the participants were chronic stroke outpatients who typically receive only one physiotherapy and possibly one occupational therapy session per week, a more intensive and structured program was considered essential to achieve measurable improvements in walking ability.

### 2.5 Statistical analysis

All data were analyzed using non-parametric statistical methods due to the small sample size and the absence of normal distribution as assessed using the Shapiro–Wilk test. To compare repeated measurements across multiple time points, the Friedman test was applied. Where significant differences were detected, post-hoc pairwise comparisons were conducted using the Wilcoxon signed-rank test. To control for multiple comparisons, Bonferroni correction was applied.

User feedback collected via the User Satisfaction Evaluation Questionnaire (USEQ) was analyzed descriptively. For each item, the mean, standard deviation, and range (minimum to maximum) were calculated.

All statistical analyses were performed using IBM SPSS Statistics for Windows, version 30.0 (IBM Corp., Armonk, NY, USA). A significance level of *p* < 0.05 was applied to all tests.

## 3 Results

### 3.1 Performance in the 10-meter walk test and the 2-min walk test

The results of the primary outcome measures are presented with and without activation of the FR by the Incedo® device. Group data are presented for four measurement time points (T0, T1, T2, and T3). Data from the follow-up 1 year after gait training (T4) are available for only four participants and are shown in Table 1, as one patient’s data are missing at this time point.

**Table 1:** Performance in the 10-meter and 2-min walk tests with and without FR activation across five time points, including one-year follow-up data for four patients.

Parameters	T0 (*n* = 4)	T1 (*n* = 4)	T2 (*n* = 4)	T3 (*n* = 4)	T4 (*n* = 4)
10mWT with activation of the FR (s)	10.9 ± 4.1	10.6 ± 4.4	8.8 ± 2.9	8.7 ± 3.1	9.0 ± 2.4
10mWT without activation of the FR (s)	11.7 ± 4.1	11.7 ± 3.9	9.6 ± 2.8	10.3 ± 3.7	9.5 ± 2.0
2minWT with activation of the FR (m)	109.3 ± 26.9	109.3 ± 27.2	125.8 ± 32.1	115.8 ± 28.5	112.0 ± 28.5
2minWT without activation of the FR (m)	103.5 ± 25.3	103.3 ± 25.4	120.5 ± 31.3	107.8 ± 26.0	105.0 ± 23.8

**Figure 1:**
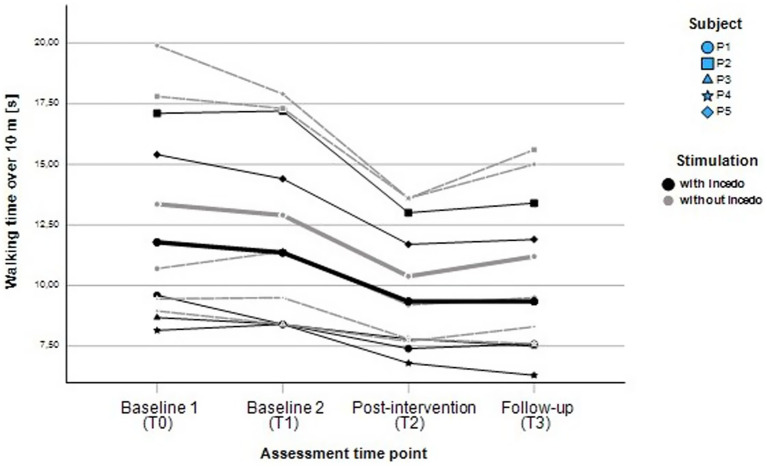
Individual patient values and mean performance in the 10-meter walk test with and without FR activation across all measurement time points in five chronic stroke patients. Individual data for each subject are displayed with thinner lines. Mean values are displayed with thicker lines. The solid line represents values with stimulation, whereas the dashed line represents values without stimulation.

Generally, two main effects were observed regarding motor performance in this study: the effect of FR stimulation during walking and the impact of gait training. The effect of FR stimulation during walking is clearly illustrated by the results of the 10mWT (Figure 1), in which increased walking speed was consistently demonstrated by patients when walking with activation of the FR compared to walking without electrostimulation across four measurement points. Although the sample size was small, a statistically significant difference was identified between walking performances with and without FR activation at the first baseline (T0) (*p* < 0.05) and during the follow-up assessment (T3) (*p* < 0.04). For the remaining two measurement sessions (T1 and T2), trends toward significance were observed (*p* = 0.07 and *p* = 0.08).

With respect to the training effects, a significant improvement in performance after gait training was observed, both with and without activation of the FR while walking (Figure 1) (*p* < 0.007). Patients required substantially less time to cover the 10-meter distance after the training compared to before the training.

In the *post-hoc* test for the measurements without activation of the FR, the following results were found: No significant difference was observed between the two baseline measurements (T0 and T1, *p* = 1.0). Significant differences were found between T1 and T2 (*p* < 0.02). No significant difference was found between T2 and T3 (*p* = 1.0).

*Post-hoc* analysis for the measurements with FR activation revealed the following results: No significant difference was found between the two baseline measurements (T0 and T1, *p* = 1.0). A significant difference was found between T1 and T2 (*p* = 0.04). No significant difference was found between T2 and T3 (*p* = 1.0).

**Figure 2:**
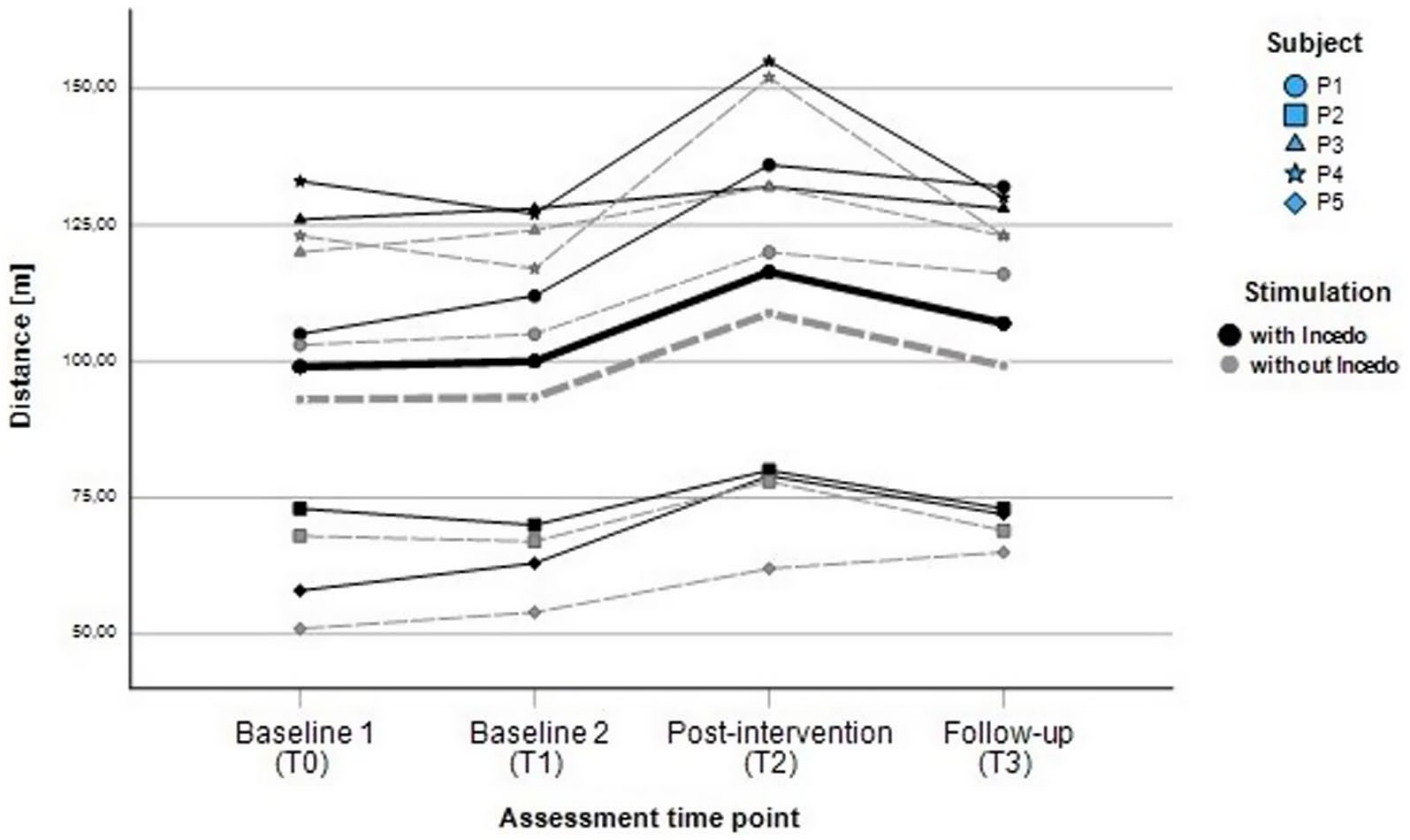
Individual patient values and mean performance in the 2-min walk test with and without FR activation across all measurement time points in five chronic stroke patients. Individual data for each subject are displayed with thinner lines. Mean values are displayed with thicker lines. The solid line represents values with stimulation, whereas the dashed line represents values without stimulation.

In the 2minWT, the same two main effects were investigated: the effect of FR stimulation during walking and the impact of gait training (Figure 2). At each measurement, the walking distance was longer when walking with activation of the FR compared to walking without it. This difference was significant at both baseline measurements (T0 and T1, *p* < 0.04) and during follow-up testing (T3, *p* < 0.04). A trend toward significance was observed in the post-testing (T2, *p* = 0.06).

Comparable to the results for the 10mWT, a significant improvement in performance after gait training was also observed in both conditions for the 2minWT (Figure 2) (*p* < 0.007). Patients thus walked substantially greater distances within the 2-min period after the training compared to before the training.

*Post-hoc* analysis of the 2minWT without FR activation revealed no significant difference between the two baseline measurements (T0 and T1, *p* = 1.0). A significant difference was found between T1 and T2 (*p* = 0.04). T2 and T3 were not significantly different (*p* = 0.8).

With FR activation, no significant difference was found between the two baseline measurements (T0 and T1, *p* = 1.0). Significant differences were observed between T1 and T2 (*p* < 0.02). No significant difference was found between T2 and T3 (*p* = 0.3).

In four patients, follow-up data including gait parameters, the 10-meter walk test, and the 2-min walk test were available 1 year after the training period (T4). Therefore, Table 1 presents data from these four patients only, including all measurement time points shown in the table. Overall, patients demonstrated improvements in both gait speed and endurance following the intervention, and these effects were largely maintained over time. In particular, gait speed assessed by the 10-meter walk test remained improved at the one-year follow-up. In contrast, the 2-min walk test showed a slight decline over time, both 3 months and 1 year after the end of training. However, despite this reduction, walking distance remained higher than baseline values measured before the intervention.

In addition to the primary outcomes focusing on walking speed and endurance, several secondary outcome measures were evaluated to gain further insight into the effects of the intervention. These included treadmill performance, user satisfaction, and gait pattern analysis based on video recordings.

### 3.2 Treadmill performance—comfortable and maximum walking speed

On average, comfortable walking speed on the treadmill improved by 0.30 km/h, from 1.92 ± 0.38 km/h before the intervention to 2.22 ± 0.34 km/h afterward (*p* = 0.06). Maximum walking speed on the treadmill increased by 0.36 km/h, from 2.90 ± 0.51 km/h to 3.26 ± 0.52 km/h (*p* = 0.07).

### 3.3 Evaluation of user experience using the USEQ

Overall, participants reported a very high level of satisfaction with the Incedo® device (Table 2).

**Table 2:** Summary of USEQ responses from the written user survey.

Question	Mean	Standard deviation	Range
Q1: Did you enjoy using the device?	4.0	0.71	3–5
Q2: Were you successful in using the device?	4.6	0.55	4–5
Q3: Were you able to control the device well?	4.2	0.84	3–5
Q4: Was the information about the device clear to you?	5.0	0.00	5–5
Q5: Did you feel discomfort while wearing the device? (inverted)	1.6	0.89	1–3
Q6: Do you think the device could help your gait rehabilitation?	4.8	0.45	4–5
Q7: Would you like to continue using the device?	4.6	0.89	3–5

Across 75 training sessions, the Incedo® system showed high technical reliability, with only one minor adverse event in which a patient perceived an unusually strong stimulus; therapy was immediately paused, the electrode was replaced, and the session was resumed without further issues, with no injury or visible skin changes/lesions (e.g., burns) at the plantar electrode site.

### 3.4 Video analysis

The blinded physiotherapist correctly identified, for all five patients, which videos were recorded before and which after the training period. For the conditions with versus without FR activation, correct classification was achieved in four out of five patients. The physiotherapist noted increased walking speed and improved walking safety both when using Incedo® and after completion of the training program. In one patient, however, the physiotherapist could not reliably distinguish between recordings with and without FR activation, as the gait pattern appeared visually almost identical across both conditions.

### 3.5 Muscle strength and sensory function results

The results of the muscle function assessment, conducted once prior to the start of the training, indicate that proximal muscle groups, such as the hip flexors and extensors, exhibited moderate to good strength (e.g., hip extension: 4.2 ± 0.8), whereas distal muscles—particularly those responsible for ankle dorsiflexion and plantarflexion—showed marked weakness (2.0 ± 0.7) (see Supplementary materials Table A). This distal impairment is clinically relevant, as it contributes to gait dysfunctions such as foot drop.

Impairments in plantar foot sensitivity were observed in all participants (see Supplementary materials Table B). Sensitivity on the affected side did not reach 100% of the values of the non-affected foot, indicating reduced somatosensory function. On the plantar surface, sensitivity was lower, with a mean value of 75.0% (SD = 16.6%) across all five patients at baseline (T2).

## 4 Discussion

This study shows for the first time in ambulatory chronic stroke outpatients, who were not enrolled in inpatient neurological rehabilitation, that gait training combined with flexor reflex activation improves walking speed and walking distance. The primary findings indicate that such an intervention is both feasible and effective in improving gait performance, with significant gains in walking speed and endurance.

### 4.1 Functional gains and sustainability

The intervention led to an approximately 25% improvement in walking speed and a 15% increase in endurance. The former remained stable over time, while the latter showed a tendency to regress during the follow-up period. This discrepancy may suggest that walking speed improvements are more likely to be retained and potentially transferred into daily ambulation habits, while endurance may require sustained or repeated training stimuli to maintain the achieved level. These findings align with earlier work suggesting that motor improvements in chronic stroke patients are possible with high-intensity and high-volume interventions (33–37).

### 4.2 Impact of flexor reflex stimulation

At all time points, gait performance with active FR stimulation surpassed that without stimulation, indicating an immediate assistive benefit of the Incedo® device. A more detailed analysis of the endurance results shows that the difference between walking with and without FR activation at the end of the five-week training phase was not significant when compared to the first baseline measurement and the follow-up measurement, meaning that the additional benefit of FR activation had at least partially diminished. Two interpretations are possible: First, with improved baseline performance due to training, the relative contribution of reflex stimulation decreased. Second, some patients may have approached their physiological performance ceiling, thereby reducing the additive value of reflex assistance.

### 4.3 Secondary outcome measures

Although treadmill performance improved modestly, the changes did not reach statistical significance. However, the trend toward faster walking speeds suggests that the intervention may also positively affect dynamic walking abilities. Specifically, comfortable treadmill walking speed increased by approximately 15.6%, and maximum walking speed improved by around 12.4% after the training period. The proportionally greater increase in walking speed during the 10mWT Test may be attributable to the inherent differences between treadmill and overground walking, given that treadmill walking is performed at an externally imposed speed, whereas overground walking is a self-paced gait activity.

The study demonstrated the technical reliability of the Incedo® system, with only one minor adverse event reported across 75 training sessions. Overall, patients tolerated the stimulation well and reported high satisfaction, as reflected by favorable USEQ scores across all domains. This high level of acceptance, together with the absence of clinically relevant side effects or discomfort, indicates that reflex-based stimulation represents a clinically viable and user-friendly technology for gait rehabilitation in ambulatory settings. Importantly, this positive safety and feasibility profile is in line with earlier reports on flexor-reflex–based stimulation (21, 22).

The blinded video analysis revealed visible changes in gait quality, including increased safety and speed, both after the training phase and during the activation of the FR, highlighting the importance of combining objective and qualitative assessments in future research.

Both muscle strength on the affected side and plantar cutaneous sensation were reduced in all patients. In future studies, these parameters should be assessed at all measurement time points to monitor their longitudinal changes over time and to better interpret and understand the effects of the training intervention.

### 4.4 Clinical implications

These findings have several implications for clinical practice. First, the results challenge the prevailing assumption that chronic stroke patients have limited potential for functional gains. With appropriate intensity and frequency, even those in the chronic phase can improve significantly. Second, the use of assistive neurotechnologies such as FR stimulation may offer an adjunct to conventional physiotherapy, especially when integrated into structured and intensive programs.

However, it is important to note that not all screened patients responded adequately to FR stimulation. In some individuals, stimulation failed to elicit a sufficient motor response or caused discomfort. Thus, appropriate patient selection remains essential. Moreover, previous reports have documented cases of stimulation-induced myoclonus, which was not observed in this study but should be monitored in future trials (22).

### 4.5 Limitations

The main limitation of this study is the small sample size (*n* = 5) and the lack of a control group. Therefore, it is not possible to isolate the specific contribution of FR stimulation from that of the gait training itself. Furthermore, we cannot rule out the possibility that patients’ positive expectations and feedback regarding improvements in performance may have contributed to a placebo effect, further emphasizing the need for a sham-stimulated control group in future studies. In addition, the study included only patients with an observable FR during screening, which may limit generalizability. Finally, no data on daily walking activity or functional use of improved gait abilities were collected, leaving open the question of ecological validity.

### 4.6 Future directions

To build upon these findings, future randomized controlled trials (RCTs) should aim to:

- Compare training effects with and without FR stimulation.
- Include larger, stratified samples to explore which patient subgroups benefit most.
- Include kinematic gait analysis to assess the effects of the intervention more objectively and in greater detail, as it enables precise and quantitative evaluation of gait parameters beyond subjective observation.
- Investigate potential neuroplastic changes using neuroimaging or neurophysiological techniques.
- Monitor walking activities before and after the treatment period.

## Data Availability

The raw data supporting the conclusions of this article will be made available by the authors, without undue reservation.
