# Supplementary material for: Activation of the flexor reflex for gait training in chronic stroke patients
Source: Front Neurol. 2026 Jul 27;17:1794512. doi: 10.3389/fneur.2026.1794512 (PMC13453826; doi:10.3389/fneur.2026.1794512)
Supplement: Supplementary file 1 [file Table_1.DOCX]

**Supplementary Materials**

**Table A** Overview of muscle strength scores in the affected lower limb at T1 (pre-training).

| **Muscle Group** | **Mean ± SD** | **Range** | **Interpretation** |
| --- | --- | --- | --- |
| Hip Flexion | 3.2 ± 0.4 | 3–4 | Moderate strength with slight variability |
| Hip Extension | 4.2 ± 0.8 | 3–5 | Mostly preserved strength |
| Hip Abduction | 3.4 ± 1.1 | 2–5 | Broader variability in strength |
| Hip Adduction | 3.6 ± 1.1 | 2–5 | Moderate strength with variability |
| Knee Flexion | 3.0 ± 0.0 | 3 | Uniform moderate strength |
| Knee Extension | 4.0 ± 1.0 | 3–5 | Generally good strength with some variability |
| Ankle Dorsiflexion | 2.0 ± 0.7 | 1–3 | Weakness, relevant for foot drop |
| Ankle Plantarflexion | 2.0 ± 0.7 | 1–3 | Weakness in distal musculature |

**Table B** Overview of plantar sensory function in the affected lower limb at T1 (pre-training).

| **Patient ID** | **Sensitivity of the sole of the foot** |
| --- | --- |
| Patient 1 | 95% |
| Patient 2 | 50% |
| Patient 3 | 80% |
| Patient 4 | 70% |
| Patient 5 | 80% |

**Individual patient data for treadmill performance at T1 (pre-training) and T2 (post-training).**

- Patient 1:
  Comfortable speed increased from 1.80 to 2.20 km/h,
  Maximum speed from 3.00 to 3.20 km/h.
- Patient 2:
  Comfortable speed improved from 1.30 to 1.60 km/h,
  Maximum speed from 2.00 to 2.40 km/h.
- Patient 3:
  Comfortable speed rose from 2.20 to 2.50 km/h,
  Maximum speed from 3.30 to 3.70 km/h.
- Patient 4:
  Comfortable speed increased from 2.30 to 2.60 km/h,
  Maximum speed from 3.40 to 4.00 km/h.
- Patient 5:
  Comfortable speed improved from 2.00 to 2.20 km/h,
  Maximum speed from 2.80 to 3.00 km/h.
